# Comprehensive Comparison of the Nutrient and Phytochemical Compositions and Antioxidant Activities of Different Kiwifruit Cultivars in Korea

**DOI:** 10.3390/plants14050757

**Published:** 2025-03-01

**Authors:** Jong-Bin Jeong, Du-Yong Cho, Hee-Yul Lee, Ae-Ryeon Lee, Ga-Yong Lee, Mu-Yeun Jang, Ki-Ho Son, Kye-Man Cho

**Affiliations:** 1 Department of GreenBio Science and Agri-Food Bio Convergence Institute, Gyeongsang National University, Naedong-ro 139-8, Jinju 528449, Gyeongsangnam-do, Republic of Korea; love_no_ri@naver.com (J.-B.J.); endyd6098@naver.com (D.-Y.C.); lar1228@naver.com (A.-R.L.); leekay11@naver.com (G.-Y.L.); jmy4330@naver.com (M.-Y.J.); sonkh@gnu.ac.kr (K.-H.S.); 2 Gyeongnam Anti-Aging Research Institute, Sancheong-gun 52215, Gyeongsangnam-do, Republic of Korea; hylee0614@gari.or.kr

**Keywords:** *Actinidia*, functional food, nutritional value, phenolic compounds, antioxidants

## Abstract

Kiwifruit is widely recognized for its rich nutritional composition and potential health benefits, yet comparative studies on different cultivars remain limited. In this study, we investigated the physicochemical properties, free sugar and organic acid content, and bioactive compounds in four kiwifruit cultivars: Hayward (HW), Halla Gold (HG), Jecy Gold (JG), and Sweet Gold (SG). This study aimed to determine variations in the composition of these cultivars and assess their antioxidant potential. The pH did not significantly differ among the kiwifruit cultivars. Sweetness and acidity are key sensory attributes in fruit, and SG exhibited the highest acidity, soluble solid content, and reducing sugar content. Accordingly, SG had the highest free sugar (11.25 g/100 mL) and organic acid (13.08 g/100 mL) levels. Phenolic acid (473.01 μg/mL) and flavonol (96.43 μg/mL) contents were most abundant in SG. In this cultivar, chlorogenic acid and epigallocatechin levels were the highest, while epicatechin and naringenin were detected only in SG. Finally, antioxidant activities (i.e., DPPH, ABTS, and hydroxyl radical scavenging activities and FRAP) were highest in SG, followed by HG, JG, and HW. The SG cultivar used in this study exhibits strong antioxidant activity, disease-suppressing effects, skin protection properties, and the potential to reduce the risk of chronic diseases due to its high phenolic compound content. These findings suggest that SG, which possesses excellent taste and functional properties, may serve as a promising candidate for the development of high-quality kiwifruit-based products.

## 1. Introduction

Recently, consumer interest in health-promoting foods has increased, and therefore, significant new research on the polyphenol and antioxidant components of plants has been conducted. Kiwifruit is popular due to its unique flavor and its richness with respect to various nutrients [1,2,3]. In addition, one kiwifruit can fulfill more than half of human daily vitamin C requirements [1,4,5,6], and so it has been reported that eating kiwis can prevent skin damage caused by ultraviolet rays and help synthesize collagen protein, a main cause of young and elastic skin [7,8]. In addition, kiwis also have numerous biologically active components, including phenols, flavonoids, vitamins E, K, Mg, Cu, folic acid, and dietary fiber. These compounds are crucial in contributing to suppressing disease, promoting health, and inhibiting digestive enzymes such as pancreatic lipase and α-glucosidase [1,6,9,10]. Finally, the peel and seeds of kiwifruit—just as much as the flesh—are rich in polyphenols and flavonoids (e.g., catechin, quercetin, and epigallocatechin), and kiwifruit seeds are rich in omega-3 fats, proteins, and antioxidants, which may contribute to the prevention of stroke and heart disease [1,6,11].

As a plant, kiwifruit is a deciduous vine that belongs to the *Actinidia* genus of the Actinidiaceae family and is widely cultivated in temperate regions. Kiwifruit was originally cultivated in southern China but is now cultivated more widely, including in China, Korea, New Zealand, the United States, Italy, and elsewhere in Europe. The kiwifruit cultivar ‘Hayward’ (*Actinidia deliciosa* cv. Hayward, HW) is cultivated both in Korea and elsewhere in the world [12,13]. In addition to *Actinidia deliciosa* (green kiwifruit), *Actinidia chinensis* (gold kiwifruit) is another class of important commercially cultivated kiwi cultivars, most of which are native to China or New Zealand [3,13]. Hayward was introduced to Korea in 1977 and has become one of the main crops in Jeollanam-do, a southeastern region of Korea, where it accounts for approximately 59% of Korean domestic production [14]. Since then, new cultivars have been developed as follows: Jecy Gold (JG) → Halla Gold (HG) → Sweet Gold (SG). Each of these is a domestic Korean cultivar with yellow flesh and is supplied and cultivated exclusively by Korean farmers [15,16,17]. JG and HG were developed by crossing *Actinidia chinensis* cv. ‘Golden Yellow’ and *A. chinensis* cv. ‘Songongu’ [15,16], while SG was obtained from a cross between *A. chinensis* cv. ‘First Emperor’ and *A. chinensis* cv. ‘Okcheon’ [17]. Kiwifruit occupies a strong position in the global fruit market for a variety of reasons, including its taste, accessibility, functionality, commercial importance, and attractive appearance. Kiwifruit products include functional foods, desserts, beverages, snacks, and cosmetics [18,19]. As new processed kiwifruit products are developed in the future, the cultivars that are most suitable may be different. Given that it is used both as a functional raw material as well as a fruit suitable for raw consumption, we have analyzed both the nutritional components of the cultivar as well as its phytochemical components.

In this study, we analyzed HW, the world’s most widely cultivated cultivar, alongside the Korean-developed cultivars HG, JG, and SG. In doing so, we quantified the relative values of these cultivars with respect to key physicochemical properties, nutrient content, the abundance of various phytochemical compounds, and their associated antioxidant activities.

## 2. Results

### 2.1. Physicochemical Properties of Different Kiwifruit Cultivars

The main physicochemical characteristics of the analyzed kiwifruit cultivars are shown in Table 1. The highest pH values were observed in cultivar HW (3.34), followed by JG, HG, and SG. Accordingly, the acidity of SG was the highest, at 2.25%, followed by HG > JG > HW. Next, the soluble solid content was the highest in SG, at 16.20°Bx, followed by HG > HW > JG. Overall, the soluble solid content was about 1.53 times higher in SG than in JG, which had the lowest content. Likewise, the reducing sugar content was the highest in SG, at 95.73 mg/g, followed by HG > HW > JG. Thus, in terms of reducing sugar content, SG was about 1.44 times higher than JG. In terms of moisture content, JG was the highest, at 90.86 g/100 g, followed by SG > HG > HW.

### 2.2. Free Sugar and Organic Acid Content of Different Kiwifruit Cultivars

The balance of free sugars and organic acids can greatly affect the sensory qualities and taste of kiwifruit [2]. In this study, we examined the free sugar and organic acid contents of different kiwifruit cultivars (Table 2). An analysis of three types of free sugars revealed that sucrose was not detected in any of the kiwi cultivars. However, the total free sugar content reached as high as 11.25 g/100 mL in SG, which was about 1.50 times higher than that of JG, which showed the lowest content. Glucose levels were the highest in SG, at 6.27 g/100 mL, followed by HG > HW > JG. The fructose level of SG was 4.98 g/100 mL, followed by HW > HG > JG. The total organic acid content was 13.58 g/100 mL in SG, followed by HG > JG > HW. Regarding the total organic acid content, SG had the highest content, which was approximately equal to 1.49 times higher than HW, which showed the lowest content. In addition, the major organic acids found in kiwifruit—i.e., succinic acid, citric acid, lactic acid, and malic acid—were found to be abundant in large amounts in all four cultivars, where they accounted for 90–95% of total organic acid content.

### 2.3. Free Amino Acid Content in Different Kiwifruit Cultivars

Amino acids are important cellular components that serve an important role in protein synthesis, metabolism, immune response, and treatment and prevention of disease [20]. The results of the free amino acid content analyses performed on the four kiwifruit cultivars are shown in Table 3. Overall, we detected twenty-seven nonessential amino acids and eight essential amino acids. The average content of nonessential amino acids (903.23 mg/100 mL) of the four kiwifruit cultivars was higher than that of essential amino acids (423.74 mg/100 mL). Moreover, among the various components, arginine was the most abundant at 162.93 mg/100 mL, followed by glutamic acid > γ-aminobutyric acid > valine > serine. In general, both arginine and glutamic acid were highly abundant (arginine: 12.28%, glutamic acid: 9.79%) and collectively accounted for approximately 22% of total amino acid content. Of the free amino acids detected, lysine, aspartic acid, arginine, glutamic acid, and GABA were the major amino acids present in the fruits [21,22,23]. The four kiwifruit cultivars used in this study also accounted for approximately 40% of total free amino acid content. The nonessential amino acid content was 1256.80 mg/100 mL in HG, followed by SG > JG > HW. The essential amino acid content was 643.17 mg/100 mL in HG, followed by SG > JG > HW. We also detected phosphoethanolamine only in SG (30.30 mg/100 mL); this compound is an important precursor of phosphatidylcholine, an important phospholipid that plays an essential role in cell membrane structure and function [24] (Table 3).

### 2.4. Phenolic Acid and Flavonol Contents of Different Kiwifruit Cultivars

Phenolic compounds play a beneficial role in preventing conditions such as diabetes, obesity, cancer, and cardiovascular disease [1,25,26]. In this study, the phenolic acid and flavonol contents of four kiwifruit cultivars were analyzed by expressing the observed values. These results are shown in Table 4. Overall, a total of nine phenolic acid compounds and six flavonol compounds were detected. The average content of total phenolic acid in the four kiwifruit cultivars was 439.99 μg/mL, of which chlorogenic acid was the most abundant at 365.66 μg/mL, followed by vanillic acid > benzoic acid > p-hydrobenzoic acid. In general, both chlorogenic acid and vanillic acid showed high content levels (chlorogenic acid: 83.11%, vanillic acid: 6.70%), and accounted for approximately 89.81% of the total phenolic acid content. Chlorogenic acid, the predominant phenolic acid compound found in the kiwifruit studied in this study, is known to be an important phenolic compound for maintaining fruit quality [27]. When comparing cultivars, SG had the highest content at 473.01 μg/mL, followed by JG > HG > HW. The average total flavonol content was 73.01 μg/mL, among which epigallocatechin was the most abundant at 43.96 μg/mL, followed by catechin > catechin gallate > epicatechin. Epigallocatechin and catechin were both highly abundant (epigallocatechin: 60.21%, catechin: 24.24%), and collectively accounting for approximately 84.45% of the total content. Epigallocatechin, the most abundant flavonol compound found in kiwifruit in this study, is known to function by reducing hyperlipidemia and exerting antibacterial activity [28]. Comparisons among cultivars showed that SG had the highest flavonol content at 96.43 μg/mL, followed by HG > JG > HW (Table 4).

### 2.5. PCA Plot and Heatmap of Primary and Secondary Metabolites in Different Kiwifruit Cultivars

**Figure 1:**
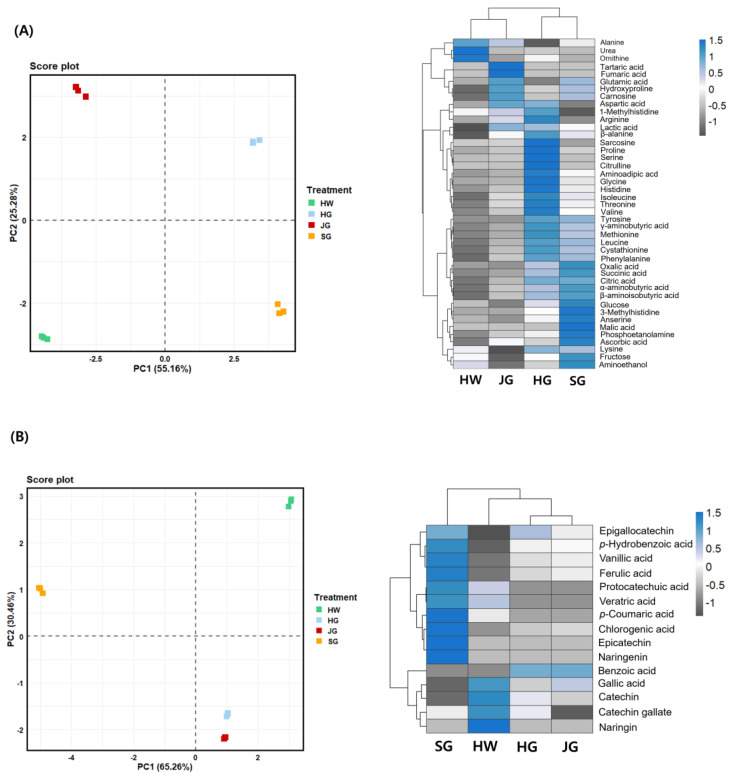
PCA plot and heatmap showing correlations between phenolic acid and flavonol contents of different kiwifruit cultivars. (**A**) PCA plot and heatmap of primary metabolites, (**B**) PCA plot and heatmap of secondary metabolites. Kiwifruit cultivars: HW, Hayward; HG, Halla Gold; JG, Jecy Gold; and SG, Sweet Gold.

PCA analysis of primary metabolites revealed that the samples can be separated into two distinct groups based on PC1 (55.16%) and further differentiated along PC2 (25.28%). Each of the four cultivars was positioned in a different quadrant, indicating distinct metabolic profiles (Figure 1A). The dendrograms at the top and left of the heatmap represent hierarchical clustering of variables and samples, respectively (Figure 1A). SG exhibited a positive correlation with free sugars and organic acids, while HG showed a positive correlation with free amino acids. SG appears to be distinct from the other cultivars, suggesting a unique metabolic profile. PCA analysis of secondary metabolites revealed that the samples were separated into two distinct groups based on PC1 (65.26%) and further differentiated along PC2 (30.46%). SG was distinctly separated along PC1, while JG and HG were located in the same quadrant, exhibiting a similar trend to the heatmap results (Figure 1B). According to the heatmap results, most phenolic acids and flavonol compounds were found at higher levels in SG compared to the other three cultivars (Figure 1B).

### 2.6. Comparison of the Antioxidant Activity of Kiwifruit Cultivars

**Figure 2:**
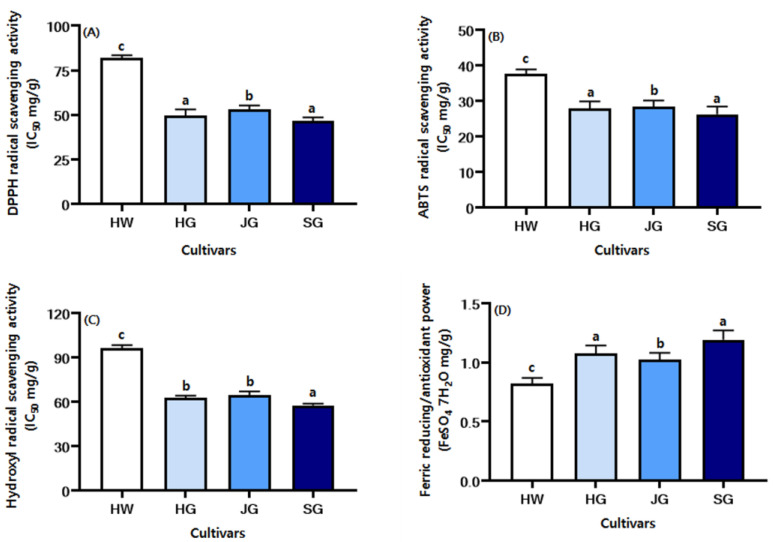
Comparison of the antioxidant activities of different kiwifruit cultivars. (**A**) DPPH radical scavenging activity, (**B**) ABTs radical scavenging activity, (**C**) hydroxyl radical scavenging activity, (**D**) ferric reducing/antioxidant power (FRAP) assay. Kiwifruit cultivars: HW, Hayward; HG, Halla Gold; JG, Jecy Gold; and SG, Sweet Gold. All values are presented as the mean ± SD of triplicate determination, and different small letters correspond to the significant differences relating to the same row using Tukey’s multiple tests (*p* < 0.05).

Kiwifruit has a relatively high antioxidant capacity among fruits [5]. Here we measured antioxidant activity in the four kiwifruit cultivars. Figure 2A shows DPPH radical scavenging activity, Figure 2B depicts ABTS radical scavenging activity, Figure 2C shows hydroxyl radical scavenging activity, and Figure 2D shows FRAP levels. The antioxidant activities, except for FRAP, were evaluated based on IC_50_ values. In general, DPPH and hydroxyl radical scavenging activity and FRAP were all measured at concentrations of 1.25, 2.5, 5.0, and 10.0 mg/mL, while ABTS radical scavenging activity was measured at concentrations of 6.125, 12.5, 25, and 50 mg/mL. For DPPH radical scavenging activity, SG exhibited the lowest IC_50_ value, at 46.48%, indicating the highest antioxidant capacity. This was followed by HG > JG > HW, and the difference in scavenging ability between SG and HW was approximately 1.76-fold. For ABTS radical scavenging activity, SG exhibited the lowest IC_50_ value, at 26.19%, indicating the highest antioxidant capacity. This was followed by HG > JG > HW, and the difference in scavenging ability between SG and HW was approximately 1.44-fold. For hydroxyl radical scavenging activity, SG exhibited the lowest IC_50_ value, at 57.51%, indicating the highest antioxidant capacity. This was followed by HG > JG > HW, and the difference in scavenging ability between SG and HW was approximately 1.67-fold. Next, we measured FRAP values to obtain more antioxidant information on kiwifruit. A compound’s reducing ability is a key measure of its potential antioxidant activity [29]. FRAP values were measured using a positive control. SG showed the highest FRAP value, at 1.19, indicating its strong antioxidant capacity. This was followed by HG > JG > HW, and the difference in scavenging ability between SG and HW was approximately 1.45-fold. Thus, regarding the DPPH radical scavenging activity, ABTS radical scavenging activity, hydroxyl radical scavenging activity, and FRAP, all cultivars showed antioxidant activity in the order SG > HG > JG > HW.

### 2.7. Correlation Between Antioxidant Activity and Phenolic Acid and Flavonol Compounds

**Figure 3:**
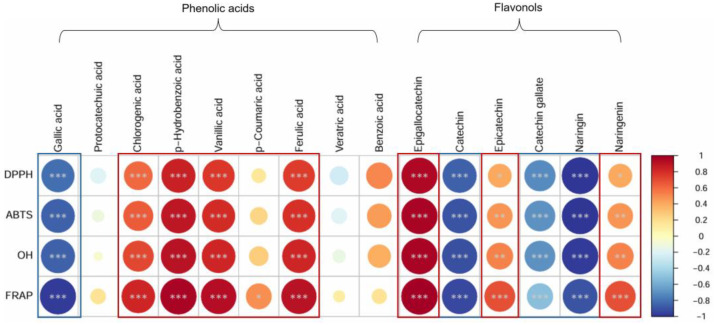
Pearson correlation coefficients between phenolic acids and flavonols analyzed in different kiwifruit cultivars and DPPH, ABTS, hydroxyl radical scavenging activity, and FRAP. *, *p* < 0.05; **, *p* < 0.01; and ***, *p* < 0.001.

Figure 3 illustrates the correlation between antioxidant activity and phenolic acids and flavonols. DPPH radical scavenging activity, ABTS radical scavenging activity, hydroxyl (OH) radical scavenging activity, and FRAP exhibited positive correlations with chlorogenic acid, p-hydroxybenzoic acid, vanillic acid, and ferulic acid, which are classified as phenolic acids, whereas they showed negative correlations with gallic acid. Among flavonols, epigallocatechin, epicatechin, and naringenin demonstrated positive correlations, while catechin, catechin gallate, and naringin exhibited negative correlations.

## 3. Discussion

In general, fruit acidity and soluble solid content play an important role in determining taste. For example, the pH of the kiwifruit reported in Zhang et al. [12] ranged from 3.16% to 4.04%, and acidity values ranged from 0.85% to 1.35%. The acidity of the four kiwifruit cultivars analyzed was 1.66–2.54 times higher. Moreover, the soluble solid content of the gold kiwifruit cultivars reported in Zhang et al. [12] ranged from 11.60°Bx to 19.13°Bx, which was similar to or higher than the values observed for SG, the cultivar that showed the highest soluble solid content. In another study, Pal et al. [2] observed that as sourness increases, sweetness typically decreases. However, we observed that the SG cultivar exhibited strong sweetness despite having a high level of sourness, suggesting that SG may possess a unique flavor profile. Addai et al. [30] found that in papaya, which undergoes five stages of ripening, pH tends to decrease while acidity and soluble solid content increase during ripening. In addition, Tehranifar et al. [31] reported that 15 pomegranate cultivars displayed a variety of physicochemical properties, implying that these properties can differ depending on fruit maturity and the specific cultivar. Taken together, these results imply that physicochemical properties vary depending on the ripening stage and cultivar type. Moreover, it has been hypothesized that the kiwifruit studied here may also show differences in physicochemical properties depending on differences in cultivar, cultivation temperature, cultivation environment, storage conditions, and soil [32].

Sugar content is one of the most important quality characteristics of kiwifruit, and sugars play a variety of roles in various plant metabolic processes [33]. In addition, fructose, which is most abundant in SG, does not react with insulin in the human body, so it is often included in the diets of diabetic patients [13]. In a previous study, Liang et al. [4] reported that the fructose and glucose contents of kiwifruit ranged from 13.06 to 33.60 mg/g. In contrast, we observed even higher levels of free sugars, although we also detected sucrose in trace amounts [4]. Fructose and glucose are the main sugars found in kiwifruit, and their abundance increases as the fruit ripens. In this study, the absence of detectable sucrose in kiwifruit may be due to the fact that the flesh had already matured and sucrose had broken down into fructose and glucose [34].

In contrast, it is known that organic acid content increases as fruits grow [35]. They are major flavor nutrients present in the fruit. Moreover, they serve an important role in human health and can delay the senescence of fruits and vegetables [36]. Dias et al. [18] reported that ascorbic acid, citric acid, and malic acid are the main organic acids found in kiwifruit. We found that SG, which had the highest organic acid content, also had higher organic acid content than the two types of kiwifruits analyzed in Dias et al. [18], with an increase of 5.06–5.24 times for malic acid, an increase of 2.87–3.53 times for citric acid, and an increase of 6.12–10.16 times for ascorbic acid. We speculate that these observed differences may be due to differences among cultivars or extraction methods. According to Zheng et al. [37], the organic acid content of peaches varies depending on the peach cultivar and ripeness level. This suggests that the organic acid content of kiwifruit may also vary depending on the cultivar and level of ripeness.

According to Song et al. [38], the free amino acid content of jujubes varied depending on maturity level. Similarly, another study by Arivalagan et al. [21] reported that the free amino acid content differed between white and red dragon fruit cultivars. Therefore, variation in the free amino acid content of kiwifruit observed in this study may be due to differences in maturity and cultivar. Compared to the results of Choi et al. [39], the green kiwifruit (HW) and gold kiwifruit (i.e., HG, JG, and SG) studied here had higher levels of free amino acid content than the green ‘Hayward’ and gold ‘Heageum’ cultivars. Similarly, the kiwifruit used in this study showed higher levels of major free amino acids than the various kiwifruit cultivars reported by Ma et al. [3]. In another study by Zhang et al. [12] the free amino acid content was found to be lowest in green kiwifruit cultivars and highest in gold kiwifruit cultivars; this finding aligns with the findings of the present study. In addition, we observed that SG and JG contain large amounts of glutamic acid, aspartic acid, and lysine, which greatly affect the flavor of kiwifruit. In particular, glutamic acid is known to enhance immune cell function [23]. Moreover, GABA, which is abundant in HG and SG, has been linked to the prevention of neurological diseases and the protection of the liver [40]. Considering all the results, it can be inferred that the diversity of kiwifruit is a factor that greatly affects free amino acid content, and kiwifruit is a source of almost all nonessential and essential amino acids.

Differences in phenolic acid and flavonol content may be caused by differences in fruit type, ripeness, and extraction method [7]. In addition, Cömert et al. [41] demonstrated that the content of phenolic acid compounds can vary depending on fruit color. When comparing chlorogenic acid content, a major phenolic acid compound found in kiwifruit, against the cultivars used by Zhang et al. [18], we found that SG exhibited significantly higher (i.e., ~14.73–35.4 times) levels. Next, while comparing p-coumaric acid and ferulic acid content, two more major phenolic acid compounds found in kiwifruit, the SG cultivar in this study was found to contain approximately 61.64–431.5 times more p-coumaric acid and 8.75–100.63 times more ferulic acid than the kiwifruit cultivars reported by Liang et al. [4]. In another study, Agatonovic-Kustrin et al. [42] found that gallic acid and chlorogenic acid were the main phenolic compounds present in mango, and their content varied depending on the cultivar. Moreover, naringenin, which was found only in SG, is a yellow crystalline powder commonly found in citrus fruits and is known to have various biological functions, including DNA protection, immune regulation, and memory enhancement [43,44]. Epicatechin was also detected only in SG, and this compound has been found to play a role in preventing diabetes, improving muscle performance, and protecting the nervous system [25]. Regarding catechin, we found that the contents of the kiwifruit cultivars studied here exceeded those reported by Hu et al. [26], who reported that kiwifruit catechin was a powerful antioxidant. The flavonol detected in this study was a type of flavan-3-ol (e.g., epicatechin and catechin) commonly detected in tea and cocoa. According to the results of Liang et al. [45], who studied grape, flavonol content depends on the cultivar. In addition, the results of Aghofack-Nguemezi and Schwab [46] on tomatoes show that flavonol content depends on the ripening stage. Thus, phenolic compound content, of which kiwi is a valuable source, can vary depending on cultivar, ripening stage, extraction method, and many other factors [47].

Reactive oxygen species (ROS) play a role in the development of various diseases, including inflammation, Alzheimer’s disease, atherosclerosis, and Parkinson’s disease [48]. ROS-induced hypoxia can be treated and prevented by increasing radical scavenging activity. Various phenolic compounds act as potent antioxidants and are considered substances with health benefits [49]. We observed that SG had a high level of phenolic compounds and correspondingly showed a high antioxidant activity. In particular, naringenin, a compound found only in the SG cultivar kiwifruit in this study, has been shown to play a direct role in scavenging reactive oxygen species and, thereby, in enhancing the activity of antioxidant enzymes [50]. A previous report by Zhu et al. [19] found that the Sungold cultivar, which has high phenolic compound content, was found to exhibit high levels of antioxidant activity. In addition, Zhang et al. [1] reported that kiwifruit peels contain more phenols and flavonoids than the flesh and, therefore, have a higher level of antioxidant capacity. Similarly, the antioxidant activity of tomatoes increased in proportion to phenol content and showed differences depending on the ripening stage and cultivar [19]. It has also been reported that citrus fruits show different antioxidant capacities depending on cultivar, origin, chemical structure, and various pre- and postharvest factors [51]. In another study, the antioxidant activity of kiwifruit showed a high correlation with phenol compound content and was therefore also affected by factors such as maturity, cultivar, and cultivation location [52]. Taken together, our results show that the kiwifruit cultivars studied here have different antioxidant activities due to the influence of various factors. Moreover, SG, which had the highest antioxidant activity, may be a potential source for the development of better kiwifruit cultivars.

This study was limited to the analysis of four kiwifruit cultivars. Future research should consider integrating aroma compound analysis using GC–MS, fatty acid profiling and enzyme inhibition assays to further elucidate the functional potential of different kiwifruit cultivars. Additionally, various analytical approaches, including untargeted metabolomics, should be employed to enable a more comprehensive comparison of the metabolic profiles among kiwifruit cultivars.

## 4. Materials and Methods

### 4.1. Kiwifruit, Reagents, and Instruments

#### 4.1.1. Preparation of Kiwifruit Cultivars

**Figure 4:**
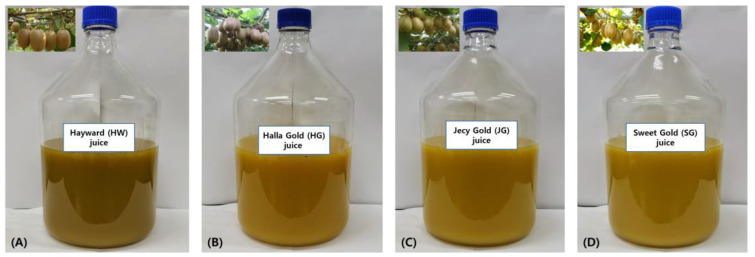
Photograph of four cultivars of kiwi fruits and their derived juices. Photograph of the fruits: National Institute of Horticultural Science and Herbal Medicine, Rural Development Administration, Jeonju-si, Jeollabuk-do, Republic of Korea. Photograph of the juices: provided by our laboratory, Jinju-si, Gyeongsangnam-do, Republic of Korea. Kiwifruit cultivars: (**A**) Hayward; (**B**) Halla Gold; (**C**) Jecy Gold; and (**D**) Sweet Gold.

HW, HG, JG, and SG cultivars used in this experiment included four kiwifruit cultivars that were supplied by Namhae-gun, Gyeongsangnam-do, of the National Horticultural Science Institute of the Rural Development Administration. The growth period varied among the cultivars, with HW requiring 176 days, JG 184 days, HG 174 days, and SG 183 days. The kiwifruit was provided at the fully ripened stage. In Namhae (Gyeongsangnam-do, Republic of Korea), where kiwifruit was cultivated, the highest recorded temperature was 37.0 °C, while the lowest was −7.7 °C. The annual average temperature was 15.1 °C, and the average precipitation was 21.05 mm. Each sample of raw material of kiwifruit cultivar (5 kg) was first washed three times under running water before being drained at room temperature for 1 h. Next, the raw material was cut into smaller sizes before being homogenized in a blender until juice was obtained; this was used in all subsequent experiments (Figure 4). Samples were then stored in a deep freezer at −80 °C and thawed as necessary to be used in later experiments.

#### 4.1.2. Reagents

Standard organic acid compounds (e.g., ascorbic acid, oxalic acid, malic acid, succinic acid, acetic acid, citric acid, tartaric acid, fumaric acid, glutaric acid, and lactic acid) and standard free sugar compounds (e.g., fructose, glucose, and sucrose) were purchased from Sigma-Aldrich (St. Louis, MO, USA). Standard phenolic acid compounds (e.g., vanillic acid, gallic acid, veratric acid, protocatechuic, p-coumaric acid, ferulic acid, chlorogenic acid, benzoic acid, p-hydrobenzoic acid, and trans-cinnamic acid) and standard flavonol compounds (e.g., epigallocatechin gallate, catechin, epicatechin, epigallocatechin, formo-noetin, catechin gallate, naringin, naringenin, vanillin, rutin, catechin gallate, DPPH (2,2-diphenyl-1-picrydrazyl), 2-thiobarbituric acid, and trichloroacetic acid ABTS (2,4,6- azin-bis (3-ethylbenzthiazoline-6-sulphnoic acid)), and TPTZ (2,4,6-tri (2-pyridyl)-1, 3,5- triazine) were obtained from Sigma-Aldrich (St. Louis, MO, USA). HPLC-grade H_2_O, methanol, acetonitrile, and glacial acetic acid were purchased from J.T. Baker (Phillipsburg, NJ, USA). All other reagents were purchased from Sigma-Aldrich.

#### 4.1.3. Instruments

pH measurements were performed using an Orion Star™ A211 pH Benchtop Meter (Thermo Fisher Scientific, Waltham, MA, USA). A Hanil micro-12 centrifuge (Seoul, Republic of Korea) was used for all experiments, as was a Saccharometer refractometer (W.S.R.O-90, Atago Co., Tokyo, Japan). Free sugar was measured using a Reflective Index (Agilent 1200 series) detector. We also used a Spectronic 2D spectrophotometer (Thermo Fisher Scientific, Waltham, MA, USA), and high-performance liquid chromatography (HPLC) was performed using an HPLC 1200 series platform (Agilent Technologies, Santa Clara, CA, USA) with a UV-DAD detector (Agilent Technologies). For gas chromatography–mass spectrometry (GC–MS), we used a GC-7890A-MSD-5975C platform (Agilent Technologies). Finally, amino acids were analyzed using a Hitachi L-8900 automatic analyzer (Tokyo, Japan).

### 4.2. Physicochemical Properties of Kiwifruit

#### 4.2.1. pH and Total Acidity

pH and total acidity were measured by modifying a previously described method [53]. Briefly, the pH of the supernatant of centrifuged kiwi juice was measured using a pH meter. Total acid was then obtained by calculating the volume of 0.1 N-NaOH that is required to be consumed to neutralize 1 mL of centrifuged supernatant to pH 8.2 ± 0.2. The total acid was then converted into equivalent amounts of lactic acid as shown in the formula below: Lactic acid (%) = 0.9 × 0.1 N-NaOH consumption (mL)/sampling volume (1 mL).

#### 4.2.2. Soluble Solids (Brix Values) and Reducing Sugars

Next, we measured the soluble solid and reducing sugar (RS) contents using a previous method with some modifications [53]. The measurements were conducted at 20 °C to ensure accuracy. We first centrifuged kiwifruit and then collected the supernatant to be measured using a refractometer. The RS was then measured by collecting a sample of the centrifuged supernatant, measuring its absorbance at 570 nm using a spectrophotometer and then interpolating it using a calibration curve.

#### 4.2.3. Moisture

The moisture content was measured using a modified version of the atmospheric pressure heating and drying method described by Dias, M. [18]. After finely pulverizing the sample, it was homogenized and used in the experiment. Moisture content was measured by the atmospheric pressure heating and drying method. A total of 3 to 5 g of the sample was placed in a weighing dish preheated to a constant weight. The lid was slightly opened and put in a dryer at a prescribed temperature for each food and dried for 3 to 5 h. It was then cooled in a desiccator for about 30 min and weighed. The weighing dish was again dried for 1 to 2 h, and the same operation was repeated until constant weight was obtained. After a drying period of 4 h, the final moisture content was determined.

### 4.3. Free Sugar Content

A free sugar analysis was performed using a previously modified method [53]. Overall, we performed free sugar analysis using HPLC. To do so, a sample of supernatant was first filtered through a sep-pak C18 column (Waters Co., Boston, MA, USA) and a 0.45 μm membrane filter (Dismic-25CS, Toyoroshikaisha, Ltd., Tokyo, Japan) after which it was used as a test solution. Next, we injected 20 μL of test solution into a sugar analysis column (Hi-Plex pb, 300 × 7.7 mm) and measured the sugar content using a Reflective Index detector while moving the mobile phase solvent water at a rate of 0.6 mL/min at 75 °C.

### 4.4. Organic Acid Analysis

An organic acid analysis was also performed using a previously modified method based on HPLC [53]. The sample was first diluted with distilled water to an appropriate concentration and then filtered through a 0.2 μm membrane filter (Dismic-25CS, Toyoroshikaisha, Ltd.) prior to organic acid analysis. Next, 20 μL of the pretreated sample was injected into an HPLC system equipped with a TSKgel ODS-100V column (4.6 × 250 mm, 5 μm, Tosoh Corp, Tokyo, Japan), and organic acid content was measured at 210 nm using a UV detector and moving the mobile phase solvent (0.1% phosphoric acid) at a rate of 1 mL/min at 30 °C.

### 4.5. Free Amino Acid Content

Free amino acid analysis was performed using a previously published method with some modifications [54]. Briefly, 1 mL of centrifuged fermentation supernatant and 4 mL of HPLC water were first mixed in a test tube, after which hydrolysis was performed at 60 °C for 1 h. Thereafter, the filtrate produced by filtering through a glass filter was concentrated under reduced pressure at 60 °C. After dissolving the concentrated sample by the addition of 2 mL of sodium citrate buffer (pH 2.2), the solution was filtered again using a 0.45 μm membrane filter before being analyzed with an amino acid automatic analyzer.

### 4.6. Phenolic Acid and Flavonol Content

Phenolic acid and flavonol analyses were performed by modifying a previously published method [55]. Here, the analysis was performed by HPLC using an Agilent 1200 platform (Agilent Technologies). Briefly, a sample of supernatant was first filtered through a 0.45 μm membrane filter (Dismic—25CS, Toyoroshikaisha) and was then used as a test solution. Next, an XBridge^TM^ C18 column (4.6 × 250 nm, 5 μm, Ireland) was used as the analytical column, and 0.2% glacial acetic acid in water (solution A) and 0.2% glacial acetic acid in acetonitrile (solution B) were used as the mobile phase solvents. Gradient conditions were based on B solvent and were as follows: 0 min: 0%, 3 min: 3%, 5 min: 5%, 8 min: 10%, 10 min: 15%, 13 min: 15%, 14 min: 3%, 15 min: 5%, 17 min: 8%, 19 min: 10%, 20 min: 15%, 22 min: 20%, 24 min: 20%, 25 min: 5%, 26 min: 15%, 27 min: 20%, 28 min: 30%, 30 min: 10%, 32 min: 40%, 35 min: 50%, 36 min: 60%, 37 min: 30%, 38 min: 40%, 40 min: 50%, 45 min: 60%, 55 min: 80%, 60 min: 90%, and 65 min: 100%. Next, 20 μL of the sample was injected, and the mobile phase speed was maintained at 1 mL/min at 30 °C. Detection was then performed using a diode array detector at UV 280 nm for phenolic acid and at UV 270 nm for flavonols (Agilent Technologies, Santa Clara, CA, USA).

### 4.7. Antioxidant Activity

The DPPH, ABTS, and hydroxyl radical scavenging activities, as well as the ferric reducing/antioxidant power (FRAP), were measured using a modified method described by Lee et al. [54], with all radical scavenging and FRAP assays performed in triplicate. To measure the DPPH scavenging activity, a 1 mM DPPH solution was prepared and adjusted to an absorbance of 0.70 at 515 nm. Subsequently, 50% ethanol extract or a positive control at various concentrations was mixed with the DPPH solution. The mixture was incubated in darkness at 20 °C for 30 min, after which the absorbance was measured at 515 nm. ABTS (7.4 mM, methanol) and potassium persulfate (2.6 mM) were mixed at a ratio of 1:1, and this reaction then proceeded for another 12 h in the dark. A prepared ABTS solution was then diluted with HPLC-grade methanol until the absorbance at 732 nm reached 0.75 ± 0.10. The prepared ABTS solution and extract were mixed at a ratio of 9:1 and reacted for three minutes before the absorbance at 732 nm was measured. The hydroxyl (OH) radical scavenging activity was measured by mixing 1400 μL of the sample with 200 μL FeSO4—EDTA solution (10 mM), 200 μL 2-deoxyribose (10 mM), and 200 μL H_2_O_2_ (10 mM); this mixture was then left to react at 37 °C for 4 h. Next, 1 mL of 1% thiobarbituric acid and 1 mL of 2.5% trichloroacetic acid were mixed in, reacted at 100 °C for 10 min, and the absorbance at 520 nm was then measured. The DPPH, ABTS, and hydroxyl radical scavenging activities were expressed as a percentage using the following equation: % = [(1 − At/Ao)] × 100. At = absorbance of kiwifruit source, Ao = absorbance of control. The ferric reducing/antioxidant power (FRAP) assay was performed using acetate buffer (30 mM, pH 3.6), TPTZ reagent (10 mM in 40 mM HCl), and FeCl3 solution (20 mM in DW). These were mixed at a ratio of 10:1:1 (*v*/*v*/*v*). After reacting at 37 °C for 15 min, 50 μL of sample and 950 μL of FRAP reagent were reacted at 37 °C for 15 min, and the resulting absorbance was measured at 590 nm.

### 4.8. Statistical Analyses

Values were calculated based on three separate tests, each of which was performed in triplicate (*n* = 3). The results of the antioxidant ratios and nutritional component levels in the samples are shown as mean ± SD. Statistical significance among sample means was assessed using ANOVA, followed by Duncan’s multiple range post-hoc tests (*p* < 0.05), performed with the Statistical Analysis System (SAS; version 9.4; SAS Institute, Cary, NC, USA). R version 4.3.3 (R Project for Statistical Computing) and R Studio (AGPL version 3) were then used for principal component analysis (PCA) and heatmap analysis [13,55]. The results of PCA were visualized using Pearson’s correlation distance method and Word’s clustering method, both of which were performed using the ‘pHeatmap’ package.

## 5. Conclusions

In this study, we performed a compositional analysis of four kiwifruit cultivars, Hayward, Halla Gold, Jecy Gold, and Sweet Gold. Sweet Gold, the most recently developed cultivar in Korea, showed higher levels of acidity, free sugar content, organic acid content, free amino acid content, phenolic acid content, flavonol content, and antioxidant activity relative to other cultivars. In addition, Sweet Gold was found to have the highest free sugar and organic acid content among the three gold kiwifruit cultivars studied here. Overall, the Sweet Gold cultivar displays a balanced sourness and sweetness and has a superior taste to other cultivars. Finally, Sweet Gold has a high phenol compound content and thus shows excellent antioxidant activity. A comparative analysis of domestic green and gold kiwifruit cultivars indicates that the Sweet Gold cultivar, which exhibits superior taste and functional properties, could serve as a potential candidate for the development of high-quality kiwifruit-based products. Further research should be conducted to validate this potential.

## Figures and Tables

**Table 1:** Measured physicochemical properties of different kiwifruit cultivars.

Contents ^1^	Cultivars ^2^			
	HW	HG	JG	SG
pH	3.34 ± 0.02 ^a^	3.26 ± 0.03 ^b^	3.31 ± 0.06 ^a^	3.23 ± 0.02 ^b^
Acidity (%, lactic acid)	2.16 ± 0.02 ^c^	2.21 ± 0.06 ^a^	2.19 ± 0.04 ^b^	2.25 ± 0.04 ^a^
Soluble solids (°Bx)	12.20 ± 0.24 ^c^	13.60 ± 0.13 ^b^	10.60 ± 0.21 ^d^	16.20 ± 0.48 ^a^
Reducing sugars (mg/g)	78.36 ± 0.78 ^c^	86.12 ± 3.44 ^b^	66.64 ± 2.00 ^d^	95.73 ± 1.91 ^a^
Moisture (g/100 g)	89.12 ± 1.08 ^c^	89.88 ± 1.02 ^b^	90.86 ± 1.10 ^a^	90.22 ± 1.04 ^a^

^1^ All values are presented as the mean ± SD of triplicate determination, and different small letters correspond to the significant differences relating to the same row using Tukey’s multiple tests (*p* < 0.05). ^2^ Kiwifruit cultivars: HW, Hayward; HG, Halla Gold; JG, Jecy Gold; and SG, Sweet Gold.

**Table 2:** Measured free sugar and organic acid contents of different kiwifruit cultivars.

Contents ^1^ (g/100 mL)	Cultivars ^2^			
	HW	HG	JG	SG
Free sugar				
Glucose	4.67 ± 0.08 ^c^	5.20 ± 0.06 ^b^	4.38 ± 0.13 ^d^	6.27 ± 0.17 ^a^
Fructose	3.82 ± 0.04 ^b^	3.80 ± 0.04 ^b^	3.13 ± 0.03 ^c^	4.98 ± 0.21 ^a^
Total	8.49	9.00	7.51	11.25
Organic acids				
Oxalic acid	0.37 ± 0.01 ^c^	0.33 ± 0.01 ^b^	0.16 ± 0.02 ^c^	0.38 ± 0.01 ^a^
Tartaric acid	nd ^3^	nd	0.38 ± 0.04 ^a^	nd
Malic acid	1.71 ± 0.01 ^d^	2.24 ± 0.04 ^b^	1.86 ± 0.04 ^c^	3.04 ± 0.05 ^a^
Ascorbic acid	0.14 ± 0.01 ^d^	0.30 ± 0.01 ^c^	0.43 ± 0.03 ^b^	0.63 ± 0.03 ^a^
Lactic acid	3.09 ± 0.03 ^d^	3.41 ± 0.05 ^b^	3.65 ± 0.03 ^a^	3.12 ± 0.06 ^c^
Citric acid	2.38 ± 0.02 ^c^	4.58 ± 0.03 ^a^	2.13 ± 0.03 ^c^	4.48 ± 0.02 ^b^
Succinic acid	1.11 ± 0.04 ^c^	1.59 ± 0.08 ^b^	1.17 ± 0.02 ^c^	1.73 ± 0.01 ^a^
Fumaric acid	nd	nd	tr ^4^	nd
Total	8.8	12.45	9.78	13.08

^1^ All values are presented as the mean ± SD of triplicate determination, and different small letters correspond to the significant differences relating to the same row using Tukey’s multiple tests (*p* < 0.05). ^2^ Kiwifruit cultivars: HW, Hayward; HG, Halla Gold; JG, Jecy Gold; and SG, Sweet Gold. ^3^ nd: not detected. ^4^ tr: ≤0.001.

**Table 3:** Measured free amino acid contents of different kiwifruit cultivars.

Contents ^1^ (g/100 mL)	Cultivars ^2^			
	HW	HG	JG	SG
Nonessential amino acids				
Taurine	nd ^3^	nd	nd	nd
Phosphoethanolamine	nd	nd	nd	30.30 ± 0.13 ^a^
Urea	172.44 ± 2.20 ^a^	14.56 ± 0.08 ^b^	nd	nd
Proline	1.08 ± 0.02 ^d^	91.38 ± 0.34 ^a^	11.26 ± 0.08 ^c^	15.21 ± 0.17 ^b^
Aspartic acid	40.24 ± 0.06 ^c^	76.34 ± 0.10 ^b^	80.51 ± 0.28 ^a^	33.01 ± 1.52 ^d^
Serine	30.24 ± 0.03 ^d^	172.42 ± 0.08 ^a^	36.83 ± 0.02 ^b^	33.21 ± 0.01 ^c^
Glutamic acid	76.94 ± 0.69 ^c^	50.61 ± 0.41 ^d^	209.17 ± 1.88 ^a^	182.65 ± 1.01 ^b^
Sarcosine	4.01 ± 0.03 ^c^	25.68 ± 0.14 ^a^	4.74 ± 0.04 ^b^	nd
Aminoadipic acid	4.43 ± 0.04 ^d^	36.48 ± 0.20 ^a^	6.86 ± 0.06 ^c^	17.51 ± 0.01 ^b^
Glycine	13.95 ± 0.13 ^d^	93.01 ± 0.51 ^a^	18.00 ± 0.16 ^c^	34.09 ± 0.19 ^b^
Alanine	73.78 ± 0.66 ^a^	8.68 ± 0.05 ^d^	59.47 ± 0.16 ^b^	43.98 ± 0.19 ^c^
Citrulline	nd	14.71 ± 0.09 ^a^	nd	nd
α-aminobutyric acid	5.30 ± 0.05 ^d^	14.56 ± 0.08 ^b^	8.99 ± 0.08 ^c^	17.45 ± 0.96 ^a^
Cystine	nd	nd	nd	nd
Cystathionine	10.52 ± 0.09 ^d^	22.11 ± 0.12 ^a^	13.78 ± 0.12 ^c^	19.32 ± 0.11 ^b^
Tyrosine	32.43 ± 0.26 ^c^	60.43 ± 0.16 ^a^	32.92 ± 0.27 ^c^	56.42 ± 0.17 ^b^
β-alanine	8.02 ± 0.06 ^c^	15.49 ± 0.03 ^a^	12.34 ± 0.10 ^b^	12.85 ± 0.03 ^b^
β-aminoisobutyric acid	19.44 ± 0.17 ^d^	33.38 ± 0.08 ^b^	24.56 ± 0.20 ^c^	36.43 ± 0.08 ^a^
γ-aminobutyric acid	30.96 ± 0.24 ^d^	241.39 ± 0.54 ^a^	63.20 ± 0.50 ^c^	179.53 ± 0.40 ^b^
Aminoethanol	9.68 ± 0.07 ^b^	8.93 ± 0.02 ^c^	7.72 ± 0.06 ^d^	11.10 ± 0.02 ^a^
Hydroxyproline	3.70 ± 0.02 ^d^	5.07 ± 0.01 ^c^	7.01 ± 0.06 ^a^	6.47 ± 0.01 ^b^
Ornithine	3.98 ± 0.03 ^a^	2.84 ± 0.02 ^b^	2.27 ± 0.02 ^c^	2.38 ± 0.01 ^c^
1-Methylhistidine	2.54 ± 0.02 ^c^	4.67 ± 0.01 ^a^	3.35 ± 0.02 ^b^	nd
3-Methylhistidine	nd	0.22 ± 0.00 ^b^	nd	0.66 ± 0.01 ^a^
Anserine	7.43 ± 0.05 ^c^	12.49 ± 0.02 ^b^	5.69 ± 0.05 ^d^	21.19 ± 0.05 ^a^
Carnosine	9.37 ± 0.07 ^d^	12.45 ± 0.02 ^c^	18.36 ± 0.15 ^a^	16.64 ± 0.04 ^b^
Arginine	114.99 ± 0.92 ^c^	253.47 ± 0.57 ^a^	180.86 ± 1.46 ^b^	102.38 ± 0.23 ^d^
Total	675.47	1256.80	807.88	872.78
Essential amino acids				
Threonine	31.96 ± 0.17 ^d^	94.18 ± 0.51 ^a^	51.05 ± 0.27 ^c^	56.48 ± 0.30 ^b^
Valine	45.31 ± 0.24 ^d^	148.98 ± 0.80 ^a^	69.49 ± 0.37 ^c^	87.93 ± 0.47 ^b^
Methionine	13.86 ± 0.07 ^d^	45.93 ± 0.25 ^a^	18.92 ± 0.10 ^c^	35.93 ± 0.19 ^b^
Isoleucine	24.96 ± 0.13 ^d^	84.57 ± 0.45 ^a^	47.41 ± 0.25 ^c^	58.49 ± 0.31 ^b^
Leucine	35.66 ± 0.12 ^d^	92.86 ± 0.33 ^a^	48.58 ± 0.17 ^c^	83.20 ± 0.29 ^b^
Phenylalanine	31.66 ± 0.11 ^d^	86.79 ± 0.30 ^a^	50.83 ± 0.18 ^c^	77.39 ± 0.27 ^b^
Lysine	48.55 ± 0.17 ^c^	59.14 ± 0.21 ^a^	22.77 ± 0.08 ^d^	55.94 ± 0.20 ^b^
Histidine	16.08 ± 0.06 ^d^	30.72 ± 0.11 ^a^	18.53 ± 0.06 ^c^	20.79 ± 0.07 ^b^
Total	8.8	12.45	9.78	13.08

^1^ All values are presented as the mean ± SD of triplicate determination, and different small letters correspond to the significant differences relating to the same row using Tukey’s multiple tests (*p* < 0.05). ^2^ Kiwifruit cultivars: HW, Hayward; HG, Halla Gold; JG, Jecy Gold; and SG, Sweet Gold. ^3^ nd: not detected.

**Table 4:** Measured phenolic acid and flavonol contents of different kiwifruit cultivars.

Contents ^1^ (μg/mL)	Cultivars ^2^			
	HW	HG	JG	SG
Phenolic acids				
Gallic acid	6.03 ± 0.02 ^a^	5.73 ± 0.02 ^b^	5.89 ± 0.02 ^a^	5.55 ± 0.02 ^c^
Protocatechuic acid	1.68 ± 0.01 ^b^	nd	nd	2.85 ± 0.03 ^a^
Chlorogenic acid	338.18 ± 1.07 ^c^	364.68 ± 1.15 ^b^	374.59 ± 1.18 ^a^	385.19 ± 1.53 ^a^
p-hydrobenzoic acid	5.39 ± 0.20 ^c^	11.08 ± 0.03 ^b^	11.39 ± 0.04 ^b^	17.16 ± 0.05 ^a^
Vanillic acid	22.54 ± 0.07 ^c^	28.15 ± 0.09 ^b^	28.92 ± 0.09 ^b^	38.22 ± 0.12 ^a^
p-coumaric acid	2.16 ± 0.01 ^b^	nd	nd	8.05 ± 0.02 ^a^
Ferulic acid	5.87 ± 0.01 ^c^	6.80 ± 0.01 ^b^	6.99 ± 0.02 ^b^	8.63 ± 0.02 ^a^
Veratric acid	4.92 ± 0.01 ^b^	nd	nd	7.36 ± 0.02 ^a^
Benzoic acid	nd ^3^	27.61 ± 0.06 ^b^	28.36 ± 0.06 ^a^	nd
t-cinnamic acid	nd	nd	nd	nd
Total	386.76	444.06	456.13	473.01
Flavonols				
Epigallocatechin	nd	62.85 ± 0.94 ^b^	42.97 ± 0.40 ^c^	70.02 ± 1.06 ^a^
Catechin	34.99 ± 0.33 ^a^	19.28 ± 0.29 ^b^	14.52 ± 0.11 ^c^	2.00 ± 0.03 ^d^
Epicatechin	nd	nd	nd	13.35 ± 0.35 ^a^
Epigallocatechin gallate	nd	nd	nd	nd
Vanilin	nd	nd	nd	nd
Rutin	nd	nd	nd	nd
Catechin gallate	8.27 ± 0.08 ^a^	6.33 ± 0.09 ^b^	3.74 ± 0.03 ^d^	5.82 ± 0.09 ^c^
Naringin	2.66 ± 0.02 ^a^	nd	nd	nd
Quercetin	nd	nd	nd	nd
Naringenin	nd	nd	nd	6.24 ± 0.20 ^a^
Total	45.93	88.45	61.23	96.43

^1^ All values are presented as the mean ± SD of triplicate determination, and different small letters correspond to the significant differences relating to the same row using Tukey’s multiple tests (*p* < 0.05). ^2^ Kiwifruit cultivars: HW, Hayward; HG, Halla Gold; JG, Jecy Gold; and SG, Sweet Gold. ^3^ nd: not detected.

## Data Availability

Data are contained within the article.
